# Supplementary material for: Electron Attachment to 5-Fluorouracil: The Role of Hydrogen Fluoride in Dissociation Chemistry
Source: Int J Mol Sci. 2022 Jul 28;23(15):8325. doi: 10.3390/ijms23158325 (PMC9369043; doi:10.3390/ijms23158325)
Supplement: Supplementary file 1 [file ijms-23-08325-s001.zip › ijms-1820302-SI.pdf]

Supporting Information
for
Electron Attachment to 5-Fluorouracil: The Role of Hydrogen Fluoride in Dissociation Chemistry

Eugene Arthur-Baidoo,[a,b] Gabriel Schöpfer,[a] Milan Ončák,[*,a] Lidia Chomicz-Mańka,[c] Janusz Rak,[c] Stephan Denifl[*,a,b]

[a] *Institut für Ionenphysik und Angewandte Physik, Leopold-Franzens Universitat Innsbruck, Technikerstrasse 25, A-6020 Innsbruck, Austria.*
[b] *Center for Molecular Biosciences Innsbruck, Universität Innsbruck, Technikerstrasse 25, A-6020 Innsbruck, Austria*
[c] *Laboratory of Biological Sensitizers, Physical Chemistry Department, Faculty of Chemistry, University of Gdańsk, Wita Stwosza 63, 80-308 Gdańsk, Poland*

*Correspondence: Milan.Oncak@uibk.ac.at; Stephan.Denifl@uibk.ac.at

Cartesian coordinates of structures optimized at the B3LYP/aug-cc-pVDZ level (in Å) along with zero-point corrected energies (in Hartree)

```
[FU-HF]-
E = -413.590298
N -0.000000 -0.907578 -0.000000
C 1.302215 -0.376872 -0.000000
C 1.232246 1.048991 0.000000
C 0.051206 1.741966 0.000000
N -1.176554 1.164694 0.000000
C -1.227598 -0.201374 0.000000
O 2.291194 -1.130380 -0.000000
O -2.277416 -0.869056 0.000000
H -0.075002 -1.919037 -0.000000
H 0.052231 2.838439 0.000000

F-
E = -99.870373
F 0.000000 0.000000 0.000000

[FU-F], iso1
E = -414.112284
N -0.000000 -0.958028 -0.000000
C 1.320529 -0.436542 0.000000
C 1.284345 1.012701 0.000000
C 0.167805 1.749794 0.000000
N -1.064788 1.116543 -0.000000
C -1.207003 -0.269679 -0.000000
O 2.285353 -1.177591 0.000000
O -2.295949 -0.817208 -0.000000
H 0.152322 2.839256 0.000000
H -1.928807 1.642444 -0.000000
H -0.079291 -1.970562 -0.000000

[FU-F], iso2
E = -414.143141
C -1.235219 0.326488 -0.000000
N 0.000000 0.982742 0.000000
C 1.246554 0.372866 0.000000
C 1.172207 -1.087844 0.000000
C -0.083723 -1.718360 -0.000000
N -1.242933 -1.082201 -0.000000
O 2.293505 1.009894 0.000000
O -2.274082 0.964511 -0.000000
H -0.134858 -2.811096 -0.000000
H -0.026247 1.997870 0.000000
H 2.107347 -1.644701 0.000000

CN-
E = -92.868847
C 0.000000 0.000000 -0.637193
N 0.000000 0.000000 0.546165

NCCH-
E = -131.495909
N -0.137162 1.278203 0.000000
C 0.000000 0.085675 0.000000
C -0.010223 -1.293548 -0.000000
H 1.021473 -1.700180 -0.000000

NCO-
E = -168.147819
O 0.000000 0.000000 1.162580
C 0.000000 0.000000 -0.070726
N 0.000000 0.000000 -1.268040

HF…NCC-
E = -231.342557
C 0.062429 0.235446 2.614765
C 0.030185 0.113893 1.263857
N 0.001421 0.005641 0.059661
H -0.035437 -0.133828 -1.485299
F -0.058911 -0.222410 -2.467118
```

HF…NCCH-
E = -231.983940
C -2.511271 -0.094272 0.001438
C -1.146401 -0.012232 -0.000409
N 0.047221 -0.104583 -0.001634
H -2.996900 0.897891 -0.000894
H 1.567669 -0.009275 -0.000955
F 2.560524 0.053609 0.000790

NCO-…HF
E = -268.633438
C -1.088703 0.088811 -0.000012
N -2.132562 -0.476632 0.000005
O -0.008392 0.709322 0.000001
H 1.329583 0.048400 0.000074
F 2.244190 -0.324380 -0.000005

HCOCHCN
E = -245.412154
H 0.762152 1.505829 -0.000000
C 1.067596 0.439199 -0.000000
C 0.000000 -0.549932 0.000000
C -1.348289 -0.182953 0.000000
N -2.476109 0.140569 -0.000000
H 0.268644 -1.607519 0.000000
O 2.248265 0.109977 0.000000

OCCCN-
E = -244.321544
C -1.108275 -0.485280 -0.000000
C -0.000000 0.106369 0.000000
C 1.267736 0.575604 0.000000
O -2.195518 -1.024160 -0.000000
N 2.372483 1.001874 0.000000

[FU-HF-CO]-
E = -300.224993
N -1.059987 0.211880 -0.000000
C -0.000000 -0.734243 0.000000
N 1.182184 -0.014346 0.000000
C 0.798913 1.276376 -0.000000
C -0.638060 1.520139 -0.000000
H 1.551844 2.067992 -0.000000
O -0.168482 -1.967107 0.000000
H -2.024484 -0.087503 0.000000

OCHCFCN-
E = -344.772948
C -0.762256 -1.004975 0.000000
C -0.000000 0.166810 -0.000000
C 1.400283 0.221894 -0.000000
O -2.009053 -1.132799 0.000000
H -0.104967 -1.912908 0.000000
F -0.632155 1.413757 0.000000
N 2.576947 0.278444 -0.000000

HF…OCCCN-
E = -344.798875
C 0.000000 -0.506312 0.000000
O -1.020077 -1.187710 0.000000
C 1.061310 0.145286 0.000000
C 2.181049 0.909074 0.000000
N 3.160724 1.566813 0.000000
H -2.497619 -0.696080 0.000000
F -3.435665 -0.450913 0.000000

CONHCONH-
E = -337.395541
N -0.180126 -1.782980 0.000000
C -0.850292 -0.656752 0.000000
N -0.000000 0.553334 0.000000
C 1.328109 0.627748 0.000000
O 2.054503 1.604679 0.000000
H -0.538764 1.419234 0.000000
O -2.078016 -0.368243 0.000000
H -0.879152 -2.529179 0.000000

OCNH2
E = -169.236218
N 0.976139 -0.481415 -0.000000
H 1.941728 -0.195086 -0.000000
C -0.000000 0.441932 0.000000
O -1.191561 0.298606 0.000000
H 0.757785 -1.475452 -0.000000

HCONH
E = -169.205595
C -0.080971 0.429860 0.044279
N 1.170530 -0.102097 -0.054097
O -1.086927 -0.288565 -0.021951
H 1.136517 -1.087243 0.246508
H -0.148983 1.531278 0.042105

HCCF
E = -176.543761
C 0.000000 0.000000 0.094151
C 0.000000 0.000000 1.298588
H 0.000000 0.000000 2.367111
F 0.000000 0.000000 -1.191505

FU, TS for ring opening
E = -514.013576
n 0.856295 -0.998084 0.484155
c -0.212698 1.182523 -0.188050
c -1.003053 0.171346 -0.185821
o -1.946578 -0.582573 -0.256645
h -3.507095 -0.274507 0.003944
f -4.446338 -0.133264 0.134540
c 1.103233 1.639360 0.031772
n 2.198463 0.937017 0.159272
c 2.054740 -0.467219 0.073402
o 3.014612 -1.177034 -0.271867
h 0.705946 -1.906262 0.036397
h 1.237281 2.728415 0.085096

FU, LM after ring opening
E = -514.083422
n 0.948458 -0.759389 -0.000135
c 0.103918 0.373165 -0.000090
c -1.280464 0.408370 -0.000004
o -2.147386 -0.473773 0.000057
h -3.705592 -0.236588 0.000020
f -4.666684 -0.141448 0.000007
c 1.024190 1.448897 0.000011
n 2.292587 1.059852 0.000043
c 2.280014 -0.348294 0.000011
o 3.254345 -1.109272 0.000049
h 0.656107 -1.723577 0.000144
h 0.740709 2.501488 0.000004

CO2
E = -188.602649
C 0.000000 0.000000 0.000000

O 0.000000 0.000000 1.167328
O 0.000000 0.000000 -1.167328

CO
E = -113.325301
C 0.000000 0.000000 -0.648027
O 0.000000 0.000000 0.486021

FU, HF with H-bond to O
E = -514.072482
C -1.888996 -0.328758 0.000229
N -0.596385 -0.911575 -0.000492
C 0.623631 -0.244304 -0.000589
C 0.437595 1.155034 -0.000475
C -0.808663 1.733553 -0.000129
N -1.965865 1.038451 0.000141
O 1.688665 -0.921841 -0.000856
O -2.865787 -1.091968 0.000823
H -0.565971 -1.925463 -0.000365
H -0.914048 2.824037 -0.000137
H 3.037384 -0.279834 0.000250
F 3.957107 0.078939 0.000973

FU, H and F coordinated to the same C-atom
E = -514.082124
C 1.545571 0.052381 -0.075177
N 0.515714 1.034585 -0.092347
C -0.812463 0.850710 0.213248
C -1.191557 -0.589369 0.336946
C -0.069040 -1.455272 0.637892
N 1.214966 -1.191995 0.363075
O -1.593293 1.799073 0.360192
F -1.792542 -0.952788 -1.094485
O 2.685521 0.416437 -0.415355
H 0.819394 1.984674 -0.269899
H -2.089225 -0.736117 0.941772
H -0.284940 -2.446382 1.047248

FU-
E = -514.061296
N 0.653580 1.043754 0.070633
C -0.788232 0.858939 0.012623
C -1.141586 -0.494672 -0.000845
C -0.237076 -1.576028 -0.062848
N 1.145246 -1.212100 0.026162
C 1.623997 0.071931 0.012647
O -1.500351 1.894679 -0.026294
F -2.487343 -0.815213 -0.018521
O 2.840535 0.330269 -0.043462
H 0.969824 2.002545 0.048708
H 1.853062 -1.907044 -0.157128
H -0.492674 -2.540759 0.386146

F pre-dissociation, TS
E = -514.027757
f -3.107351 -0.827770 -0.246790
c -1.199362 -0.124869 0.204282
n 0.885935 -1.342537 0.083709
c -0.514845 -1.281000 0.194921
h -1.013303 -2.249073 0.254372
h 1.377149 -2.225640 0.086835
c 1.677478 -0.228041 -0.048709
c -0.464725 1.119201 0.073204
n 0.957220 0.946653 -0.056637
h 1.505175 1.795250 -0.147933
o 2.910513 -0.275715 -0.149417
o -0.885040 2.274319 0.061435

FU
E = -514.042586
C 0.000000 1.165935 -0.000000
C 1.210538 0.342192 0.000000
C 1.152669 -1.004859 0.000000
N -0.066125 -1.653521 -0.000000
C -1.291076 -0.997836 -0.000000
N -1.171513 0.386346 -0.000000
O -0.040303 2.382102 -0.000000
O -2.358782 -1.585121 -0.000000
H -2.046826 0.899827 -0.000000
H -0.114718 -2.663000 -0.000000
H 2.046209 -1.624910 0.000000
F 2.393189 0.983319 0.000000

[FU-H]-
E = -513.523172
C 0.000000 -1.140524 0.000000
C 1.175545 -0.323540 -0.000000
C 1.085293 1.049552 -0.000000
N -0.051729 1.771284 -0.000000
C -1.227237 1.076450 -0.000000
N -1.144593 -0.338093 0.000000
O -0.077636 -2.381141 0.000000
O -2.359942 1.588979 -0.000000
H -2.030507 -0.829398 0.000000
H 2.021897 1.620236 -0.000000
F 2.409098 -0.939723 -0.000000

[FU-H]-, HF next to O
E = -513.453997
N 2.011550 1.124380 -0.000007
C 1.942017 -0.284001 -0.000018
N 0.628184 -0.809242 -0.000002
C -0.602230 -0.102069 0.000033
C -0.465280 1.340122 0.000019
C 0.796508 1.506384 0.000004
O 2.919092 -1.031092 -0.000019
O -1.644230 -0.798737 0.000051
F -4.022298 -0.028371 -0.000032
H 0.568039 -1.818319 0.000009
H -3.070477 -0.256287 -0.000137

[FU-H]-, HF next to H
E = -513.451002
N 1.073272 0.072178 0.000000
C -0.000000 0.989481 -0.000000
N -1.287271 0.411124 -0.000000
C -1.667680 -0.967948 -0.000000
C -0.494097 -1.847357 0.000000
C 0.515685 -1.096491 0.000000
O 0.144494 2.210793 -0.000000
O -2.864157 -1.251800 -0.000000
F 3.614797 0.552175 0.000000
H -2.046685 1.078362 -0.000000
H 2.645370 0.430893 0.000000

NCH2
E = -93.971904
C -0.505771 0.000171 0.000152
N 0.742348 0.000086 -0.000041
H -1.080012 -0.945251 -0.000311
H -1.081798 0.943624 -0.000312

HCN
E = -93.420892

H 0.000000 0.000000 -1.576035
C 0.000000 0.000000 -0.501640
N 0.000000 0.000000 0.655125

HF
E = -100.451506
H 0.000000 0.000000 -0.833058
F 0.000000 0.000000 0.092562

FU, F moving around the ring, TS
E = -514.025093
n 1.258523 0.727355 -0.000001
c -0.023959 1.372511 -0.000000
c -1.104399 0.420467 -0.000003
c -0.888179 -0.904800 -0.000002
n 0.411192 -1.415333 -0.000000
c 1.539405 -0.624133 0.000000
o -0.045682 2.601202 0.000001
f -3.427326 -0.700156 0.000002
o 2.687468 -1.078951 0.000002
h 2.064773 1.341936 0.000000
h 0.577780 -2.411829 0.000001
h -1.756119 -1.575137 -0.000002

FU, F moving around the ring, LM
E = -514.027733
n -0.886175 -1.048949 -0.000000
c -1.179170 0.359536 0.000000
c 0.000000 1.203735 0.000000
c 1.211666 0.624114 0.000000
n 1.392136 -0.769736 -0.000000
c 0.347612 -1.662404 -0.000000
o -2.366655 0.677597 0.000000
f 0.704883 3.213765 0.000000
o 0.501238 -2.890446 -0.000000
h -1.685509 -1.673247 -0.000000
h 2.314821 -1.181608 -0.000000
h 2.127695 1.214674 0.000000

FU, HF formed after F moving
E = -514.076642
C 0.000000 1.013699 -0.000000
N 1.312058 0.492285 -0.000000
C 1.717198 -0.855805 0.000000
C 0.560365 -1.698336 0.000000
C -0.714507 -1.223292 0.000000
N -1.025276 0.102918 0.000000
O 2.918662 -1.153596 0.000000
O -0.151010 2.241045 -0.000000
H 2.053544 1.183956 -0.000000
H -2.487463 0.406609 0.000000
H -1.576086 -1.897190 0.000000
F -3.501892 0.447001 0.000000

H
E = -0.501657
H 0.000000 0.000000 0.000000

NCCH2…HF
E = -232.542823
C -2.486131 -0.000298 -0.000206
C -1.100892 0.000145 0.000035
N 0.071373 0.000559 0.000518
H -3.029049 0.942570 -0.000338
H -3.028493 -0.943481 -0.000335
H 1.859944 0.000187 -0.000261
F 2.802237 -0.000252 -0.000185

FU, H move, TS
E = -514.027713 -514.027713
n -1.185940 -1.222352 -0.046690
h -2.022911 -2.369814 0.653281
c 0.139925 -1.479089 -0.029275
c -1.609643 0.081569 -0.023010
n -0.591472 1.060316 0.015911
c 0.795029 0.875277 0.011620
c 1.119599 -0.525369 -0.016538
f 2.445543 -0.884503 -0.015769
o 1.575510 1.837188 0.038124
o -2.790393 0.446278 -0.034017
h -0.910064 2.022596 0.030931
h 0.414574 -2.540068 -0.016490

FU, H move, LM
E = -514.028114
N -0.062792 -1.698599 0.000000
H 0.303751 -3.572693 0.000000
C 1.086356 -0.997878 0.000000
C -1.245983 -1.015551 0.000000
N -1.151136 0.396300 0.000000
C -0.000000 1.191414 0.000000
C 1.177194 0.370863 0.000000
F 2.406218 0.989104 0.000000
O -0.070226 2.429624 0.000000
O -2.372508 -1.530894 0.000000
H -2.033458 0.894715 0.000000
H 2.007717 -1.590789 0.000000
